# B-Cell Responses to Sars-Cov-2 mRNA Vaccines

**DOI:** 10.20411/pai.v7i2.550

**Published:** 2022-12-09

**Authors:** Lela Kardava, Clarisa M. Buckner, Susan Moir

**Affiliations:** 1 Laboratory of Immunoregulation, National Institute of Allergy and Infectious Diseases (NIAID), National Institutes of Health (NIH), Bethesda, MD

**Keywords:** SARS-CoV-2, vaccination, protective immunity, B cells, plasmablasts, memory response, antibodies

## Abstract

Most vaccines against viral pathogens protect through the acquisition of immunological memory from long-lived plasma cells that produce antibodies and memory B cells that can rapidly respond upon an encounter with the pathogen or its variants. The COVID-19 pandemic and rapid deployment of effective vaccines have provided an unprecedented opportunity to study the immune response to a new yet rapidly evolving pathogen. Here we review the scientific literature and our efforts to understand antibody and B-cell responses to SARS-CoV-2 vaccines, the effect of SARSCoV-2 infection on both primary and secondary immune responses, and how repeated exposures may impact outcomes.

## INTRODUCTION

The rapid global spread of SARS-CoV-2 and its deadly consequences quickly mobilized the scientific community to develop a panoply of vaccines [1], several of which have been highly effective at preventing transmission or severe disease [2]. While the COVID-19 pandemic has caused worldwide suffering that will be felt for years to come, it has provided a few silver linings, one being an unprecedented opportunity to learn how the human immune system responds to a new pathogen and how new technologies can be rapidly translated into safe and effective vaccines. As with most vaccines, protection from SARS-CoV-2 infection or COVID-19 illness is largely mediated by antibodies, in this case against the viral spike protein delivered in the form of mRNA-based nanoparticles, adenoviral vectors, inactivated whole virus, or recombinant proteins [3]. Humoral immunity is sustained by 2 distinct arms of the B lineage, the plasma cells that secrete the antibodies and the memory B cells (MBCs) that can rapidly differentiate into antibody-secreting cells upon re-encounter with antigen. Antibody-mediated immunity can also be divided into different categories: namely primary versus secondary or recall responses, direct antigen-neutralizing and protective non-neutralizing functions, and whether a response or phase of a response is independent or not of T-cell help. This review will mainly focus on human B cells that are involved in generating and sustaining protective immunity following exposure to SARS-CoV-2 through infection and/or vaccination. Excellent reviews can be found elsewhere on the functional aspects of antibodies [4], and lineages such as B-1 and marginal zone B cells (MZB) involved in providing T-independent natural immunity [5]. This review takes a chronological approach both in terms of the different stages of the COVID-19 pandemic and how they relate to different stages of B-cell immunity.

## B CELLS ENGAGED IN THE PRIMARY IMMUNE RESPONSE TO mRNA VACCINES

### Plasmablasts in response to infection

In the early days of the COVID-19 pandemic when death rates were high, it quickly became apparent that the host response to the virus could have both disease exacerbating and beneficial effects [6–9]. Among the earliest B cells to be detected in the peripheral blood after acute infection or vaccination are plasmablasts, actively proliferating low affinity antibody-secreting cells that arise during the early extrafollicular T-independent phase of the immune response [5]. Plasmablasts are among the earliest indicators of acute infection, including with SARS-CoV-2 [10–12], in part because they are rapidly induced after exposure but also because basal levels are low and their distinctive features make their appearance in the circulation easy to track. The surge in plasmablasts may also arise from antigen nonspecific innate responses involving cytokines that are triggered by acute viral infections, including SARS-CoV-2 [13]. This acute cytokine storm includes IL-6, CXCL10, and IL-10 [14], which can induce B-cell terminal differentiation, even in the absence of antigen [15]. In HIV infection, serum levels of these cytokines correlate with frequencies of plasmablasts in the blood, of which the highest frequencies occur during early infection when viremia is high [16]. In SARS-CoV-2 infection, the immunoglobulin gene repertoire of acute-phase plasmablasts has been reported as diverse, suggesting a polyclonal rather than virus-specific induction [10]. However, in other studies, plasmablast expansions have been associated with high antiviral antibody titers suggestive of a specific response to the virus [17, 18]. The plasmablasts may contribute to the early low-maturity antibody response that declines rapidly before a more stable neutralizing response arises from long-lived bone marrow-derived plasma cells [19]. However, there is uncertainty regarding the longevity of plasma cells against SARS-CoV-2 [20], especially given the evidence of rapidly waning immunity following infection [21].

### Plasmablasts in response to vaccination

The rapid development and approval of vaccines against SARS-CoV-2 under the emergency authorization use in late 2020 provided an unprecedented opportunity to investigate immune responses to what was essentially a neoantigen and to do so with study designs that could include baseline analyses which could not be performed with SARS-CoV-2 infection. This was also the first opportunity to delineate responses to an mRNA-based vaccine in humans. For the purpose of this review, the 2-dose regimen of either the mRNA-1273 (Moderna) or the BNT162b2 (Pfizer-BioNTech) is considered a primary immunization. As with SARS-CoV-2 infection, plasmablasts are induced rapidly following vaccination, of which a fraction may reflect recall response to cross-reactive elements in the S2 subunit of coronavirus spike proteins [22]. However, in contrast to infection or other chronic conditions where plasmablasts in the blood can remain elevated for as long as the active disease persists [23], those induced by vaccination tend to arise and disappear rapidly, consistent with their short-lived nature [24, 25]. In response to a first exposure, vaccine-specific plasmablasts in the blood reach their peak nearly 2 weeks after vaccination [26], while for subsequent exposures, the peak occurs faster, within about 7 days, and the plasmablasts are more likely to originate from an MBC recall response [27]. However, plasmablast responses can vary with different types of vaccines and routes of administration [28]. A further confounder is that most studies report on total plasmablasts and not necessarily those that are specific to the vaccine.

**Figure 1.**
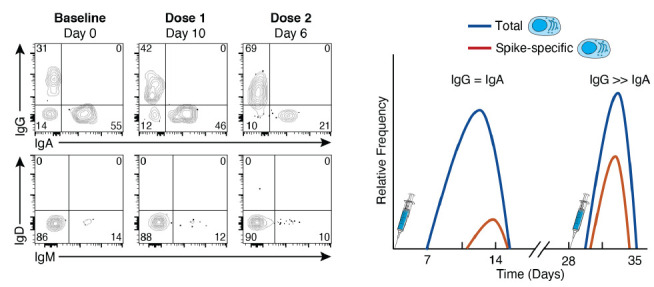
**Plasmablast responses to SARS-CoV-2 vaccination.** The flow cytometry plots are representative of immunoglobulin isotype cell-surface staining of plasmablasts at baseline and at day 10 after the first dose and day 6 after the second dose of the mRNA-1273 vaccine. The plots illustrate that IgA is the dominant isotype at baseline (steady state) while IgG dominates after vaccination, IgM remains low and stable over time, and the cumulative distribution of the 3 isotypes is ~100% at each timepoint. The graphical depiction on the right illustrates the kinetics for total (antigen non-specific) and spike-specific plasmablasts following doses 1 and 2 of the mRNA-1273 vaccine. The non-specific response precedes the spike-specific response after dose 1, while both are elicited intensely albeit more transiently after dose 2 and dominated by IgG for both spike-specific and non-specific responses.

When SARS-CoV-2 vaccines became available at the end of 2020, NIH employees who were eligible received their vaccines onsite, which happened to be the 2-dose mRNA-1273 vaccine, given 28 days apart [3]. We took this opportunity to evaluate the kinetics and magnitude of the B-cell and antibody response to this novel vaccine in a cohort of 21 NIH healthcare and laboratory employees. We used spectral flow cytometry, which captures the entire emissions spectrum and generates a spectral fingerprint for each fluorochrome [29], to evaluate both antigen-specific (the SARSCoV-2 spike protein) and non-specific B-cell responses among the major B-cell subsets that circulate in the peripheral blood, including plasmablasts, on freshly isolated samples [30]. We use the term non-specific to define responses that are not antigen-specified and note that a portion of this response may be specific but not captured by the assay used. Our design overcame 2 common challenges when studying plasmablasts: 1) the unavoidable detrimental effects of cryopreservation on their viability [31]; and 2) the inherent limitations of quantifying antigen-specific plasmablasts using quasi single parameter methods such as ELISPOT [32]. Our design also had its limitations, including the drift in lasers and settings that are difficult to normalize when performing assays in real-time over an extended period and the reduced surface expression of the B-cell receptor (BCR) that occurs as B cells differentiate into plasmablasts and increase their expression of immunoglobulins (Igs), which are destined for secretion [33]. The loss of cell surface BCR expression on plasmablasts is more pronounced for IgG than IgA, the predominant isotype at steady state (Figure 1). There is also evidence that the ratio of surface and intracellular expression of Igs differs with conditions and tissue origin [33]. Nonetheless, we and others have used flow cytometry to identify and quantify antigen-specific plasmablasts without the need to perform intracellular staining [27, 30, 34].

Several approaches can be taken to verify the experimental design; in our study we confirmed that ~95% of all plasmablasts had an accounted Ig isotype [30], IgA, IgG, or IgM (Figure 1). Verification also included the comparison of 2 methods of plasmablast quantification, one based on flow cytometry and the other by ELISPOT, as well as the comparison of isotype distribution with and without permeabilization [30].

**Figure 2.**
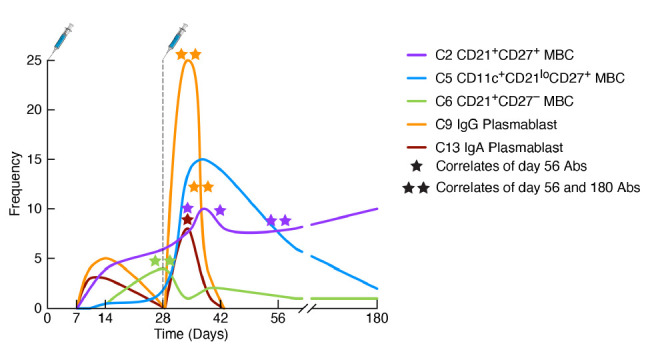
**Kinetics of spike-specific plasmablasts and memory B-cell responses to SARS-CoV-2 vaccination.** The graph depicts cellular responses over time following doses 1 and 2 of the mRNA-1273 vaccine, with corresponding cluster (C) designations and annotations discussed in the review. The stars refer to the timepoints when the indicated spike-specific plasmablast or memory B-cell (MBC) cluster correlated with either day 56/month 2 (1 star) or both day 56/month 2 and day 180/month 6 (2 stars) of the spike-specific antibody response. Both plasmablast clusters correlated with the antibody response, as did the early MBC C6 and the conventional MBC C2 but not atypical MBC C5. Abs, antibodies.

Having determined that infection or vaccine-induced SARS-CoV-2-specific plasmablasts in the blood could be quantified by spectral flow cytometry, we proceeded with an unbiased and integrated approach (explained in detail below) to longitudinally evaluate primary antibody, plasmablast, and MBC responses to the 2-dose mRNA-1273 in our cohort of 21 SARS-CoV-2-naive healthy adults [30]. A detectable rise in total (SARS-CoV-2 spike nonspecific) IgG and IgA plasmablasts was observed approximately 7 days after the first vaccine dose and peaked around day 10 (illustrated in Figure 1 and clusters C9 and C13 respectively in Figure 2). Plasmablasts that were specific for SARS-CoV-2 spike protein could be detected by day 10 and peaked after day 14, the last timepoint evaluated after dose 1 [30]. Both levels of spike-specific and non-specific plasmablasts had returned to baseline when the second dose was administered at day 28. The plasmablast response to the second dose was rapid, intense, and short-lived, with a peak occurring 5-7 days after vaccination (illustrated in Figure 1). We then addressed whether vaccine-induced plasmablasts measured in the peripheral blood were predictive of the antibody response measured in the serum. We found spike non-specific plasmablasts at days 10 and 14 after dose 1, and spike-specific plasmablasts at days 7 and 10 after dose 2 were correlated with both IgG and IgA antibody titers measured at 2 and 6 months after vaccination [30]. While few studies have established similar correlations, it may be because most studies have been performed on cryopre-served cells at relatively few timepoints. The observation that frequencies of circulating plasmab-lasts are predictors of the antibody response to SARS-CoV-2 vaccination could reflect overall immune competency and/or that plasmablasts are precursors of plasma cells that home to the bone marrow and secrete the antibodies, which are then measured in serum [35]. Recent findings on the dynamics of influenza vaccine-induced B-cell and antibody responses provide evidence for a direct link between plasmablasts, bone marrow plasma cells, and serum antibodies [36].

### Memory B cells

While the concept of immunological memory, the ability to rapidly respond to a previously encountered pathogen through the generation of affinity-matured MBCs, is straightforward, the reality of identifying and tracking MBCs is far more challenging. Since the identification almost 25 years ago of CD27 as the canonical or conventional marker of human MBCs [37, 38], several phenotypically distinct populations of MBCs have been identified [39]. Their numbers and relative distribution can vary with time, condition, and anatomical location, all of which suggest that the heterogeneity of MBCs also reflects distinct functionalities in response to pathogens [40, 41]. With the development of new technologies and computational tools that can handle the acquisition and analysis of large datasets [29], several recent studies have provided new insights into the complexity and diversity of B-cell populations, as well as clarity regarding MBCs involved in the generation of immunological memory [31, 42]. For example, while the existence of MBCs that lack expression of CD27 has been known for some time [43–45], only recently have combinations of markers such as CD200, CD45RB, CD11a, and CD11c helped discriminate between the various conventional and nonconventional human MBCs [31, 42]. When combined with analyses of BCR mutational burden, integrated approaches can also provide insight into the positioning of various MBC populations along a maturational trajectory, such as the progressive accumulation of mutations from CD27^-^CD45RB^-^ to CD27^-^CD45RB^+^ to CD27^+^CD45RB^-/+^ MBCs [31]. However, while much progress has been made, certain markers may have a more nuanced profile than has been assumed from past experience. For example, MBCs are often defined as CD38^-^ or CD38^lo^ [31, 46]. However, while transitional B cells and plasmablasts express distinctly high levels of CD38 and all naive B cells express intermediate levels of CD38, MBCs can express a wide range of CD38 intensities, from the very low intensities on nonconventional (CD11c^+^CD21^lo^CD27^+/-^) MBCs to variable yet distinct intensities on conventional (CD11c^-^CD21^+^CD27^+^) MBCs [30, 47]. Another caution is using the BCR mutational burden to assess the maturational stage of an MBC population that has a heterogeneous or partially undefined Ig isotype profile, especially given that Ig isotype is a strong determinant of mutational burden [48]. In the example given above, MBCs, defined by expression of CD27 and CD45RB, had different distributions of Ig isotype which could have contributed to the differences in mutational burdens reported [31]. Nonetheless, integrated multi-omic approaches are providing new means of classifying B cells that will help advance our understanding of MBCs, how they are generated and how they contribute to immunological memory.

In our flow cytometric approach to evaluating B-cell signatures of the primary antibody response to the 2-dose SARS-CoV-2 mRNA-1273 vaccine, we designed a panel that included markers for distinguishing between conventional and non-conventional MBCs (CD11c, CD21, and CD27), as well as the major Ig isotypes (IgD, IgM, IgG, and IgA). The reasoning for including all 4 Ig iso-types was 2-fold: to compare cellular Ig isotypes with their corresponding spike-binding antibody titers and to extend analyses of manually defined populations to unsupervised clustering that would capture a broad spectrum of phenotypes [30]. Given the potential pitfalls of using dimensional reduction and clustering algorithms to generate immune signatures [29], the inclusion of the 4 major Ig isotypes provided a means of verifying clusters based on canonical rules. The most abundant clusters should be naive B cells expressing higher intensities of IgD than IgM, and there should be few and minimally abundant clusters that contain incompatible isotypes; for example, a cluster should not contain both unswitched (IgD/M) and switched (IgG or IgA) isotypes or IgG with IgA. We also found that a 30-cluster setting was ideal for generating a wide spectrum of distinct phenotypes without compromising visual clarity or quantitative analyses. Having ascertained that our clustering met these metrics and was consistent with manually defined populations, we then evaluated the temporal dynamics of each cluster in response to the 2 vaccine doses by measuring fluctuations of each cluster (the non-specific response) and of spike-bound cells (the antigen-specific response) within each cluster [30]. This approach identified several MBC clusters that fluctuated over time after each vaccine dose, and a number of these clusters were found to correlate with antibody titers at months 2 and 6 post-vaccination. An early spike-specific correlate of both timepoints was cluster 6 (C6; illustrated in Figure 2), an IgG-switched CD38^+^ MBC lacking expression of CD27 that has been described as an early-response MBC [49]. Notably, frequencies at baseline of dose 2 of spike-specific cells in C6 correlated with the IgG and IgA antibody responses at months 2 and 6 post-vaccination. One might ask why an IgG-expressing MBC like C6 would correlate with IgA antibodies? It is likely that these correlates are indicative of an overall responsiveness rather than a cause-effect relationship. In this regard, C6 was among several clusters, both switched and unswitched MBCs, that were early (dose 1) antigen non-specific correlates of the IgG spike-specific antibody response [30].

Our study on B-cell signatures of antibody response to mRNA vaccination included several MBC subsets. Among the 30 clusters generated by the unsupervised analysis, 18 were MBCs, consistent with their heterogeneous nature and a reflection of the B-cell markers chosen to study the MBC response. Among the 18 MBC clusters, 9 were classified as conventional, as defined by MBCs that express both CD21 and CD27, then further annotated as unswitched or IgA or IgG switched, and as being either CD38^+^ or CD38^-^, consistent with another recent study [47]. The remaining 9 MBC clusters were classified as nonconventional, either because they lacked expression of CD27 or had profiles associated with activation (CD20^hi^CD21^lo^CD11c^+^). Among these were MBCs we have previously described in the context of persistent HIV viremia and named activated (CD27^+^) or tissue-like (CD27^-^) [45], although the term atypical is now commonly used to refer to both collectively [50]. Atypical MBCs accounted for 6 clusters: either unswitched or IgA or IgG switched, and among each, they were further delineated by the expression of CD27 [30]. Notably, none of these atypical MBCs expressed CD38, consistent with previous reporting [51]. Several of the atypical MBC clusters underwent spike-specific and non-specific fluctuations following vaccination. However, while several atypical MBC clusters correlated antigen-nonspecifically with antibody titers at month 2, few were spike-specific correlates, despite one of these clusters, C5, an IgG-expressing atypical MBC comprising a large fraction of the spike-specific MBC response days 7-14 after dose 2 [30], and depicted in Figure 2. Thus, it was the non-specific overall activation of atypical MBCs that was predictive of the antibody response. In contrast, a strong spike-specific MBC predictor of antibody responses at both months 2 and 6 post-vaccination was C2 (Figure 2), an IgG-expressing resting MBC that also accounted for most of the strong month 6 spike-specific MBC response that we and others have described [30, 52]. However, one important caveat to our study is that our panel did not include the activation marker CD71, which is strongly induced following vaccination or infection [27]. It will be important to include this marker in future studies as it seems to have a different expression profile than other markers of activation, such as CD11c and CD95 [31].

## SECONDARY EXPOSURE FROM INFECTION AND/OR VACCINATION

Since the start of the COVID-19 pandemic in 2020, exposures to SARS-CoV-2 have transitioned in rapid succession from primary infection and vaccination alone to secondary combined exposures, and most recently, to multiple combinations of re-infection and/or booster vaccination. Given the high transmissibility of the virus and the rapid development and distribution, albeit uneven, of vaccines, the number of people worldwide who remain unexposed to SARS-CoV-2 has been quickly diminishing. This means that there are fewer and fewer people who remain immunologically naive to the virus and conversely, there is a growing number of people with diverse types of exposures, whether from infection and/or vaccination. These changing dynamics pose both challenges and opportunities; while it is becoming increasingly difficult to understand how multiple and varied exposures shape the immune response to SARS-CoV-2, the knowledge gained may help understand what sustains protection against rapidly evolving viruses such as SARS-CoV-2. Furthermore, much of the knowledge with SARS-CoV-2 is likely to be applicable to other current and future viral pathogens.

The term hybrid refers to the type of immunity that arises from a combination of infection and vaccination, in either order. While the definition is simple, the reality of tracking and understanding hybrid immunity is far from simple. For example, should we consider infection with other coronaviruses as part of hybrid immunity given the evidence that immune responses to SARSCoV-2 can be modulated by cross-reactive B-cell and T-cell responses that were acquired prior to the pandemic [22, 53]? Exposure histories are important because they can influence the magnitude and longevity of subsequent immune responses yet are affected by factors and mechanisms that are not well understood. Contributing factors likely include the nature and timing of each exposure, and the role of imprinting, the process by which the immune system tends to amplify a response to the first version of the pathogen encountered at the expense of subsequent variants [54]. We tackle a few of these factors here.

### The effect of prior infection on responses to primary vaccination

The first reports on the effects of prior infection on the immune response to vaccination began to emerge in the summer of 2021, approximately 6 months after the first SARS-CoV-2 vaccines received emergency use approval [55]. Several studies performed during this period demonstrated that 1 or 2 doses of the mRNA vaccines augmented the magnitude and durability of neutralizing antibody titers and increased breadth among serum and memory B-cell derived antibodies in people who were previously infected when compared to infected/unvaccinated or uninfected/vaccinated individuals [56–65]. Of note, however, the second dose was found to provide minimal enhancement of antibody and B-cell responses over the first dose in previously infected individuals [58], contrasting with a strong response to a second dose in uninfected individuals [57, 62, 65]. These findings were early indications that the benefit provided by hybrid immunity may be restricted by factors associated with time interval or repeated exposure, as has been shown with other vaccines and in animal models [66–68]. A large retrospective study addressing the risk of infection also found that a second dose of the BNT162b2 mRNA vaccine in people who were previously infected did not increase protection from re-infection over the first dose [69]. Regarding time interval between doses, several studies on cohorts that have included both uninfected and previously infected individuals have shown that an extended interval between the first 2 doses of the mRNA vaccines increases the magnitude and breadth of B-cell and/or antibody responses when compared to the standard 3-to-4-week interval [62, 70–75]. However, while immunological benefits of an extended dosing interval have clearly been demonstrated, evidence for protection from infection or severe disease is more nuanced. In another large retrospective study demonstrating enhanced protection from vaccination after prior infection, a prolonged interval between vaccine doses did not contribute to added protection in either uninfected or previously infected participants [76]. However, other, albeit smaller and less definitive studies have demonstrated increased protection with extended intervals [73, 77], consistent with health-impact modeling [78], and changes in guidelines on dosing interval [79]. Several factors may contribute to differences in predicted or observed outcomes, including demographics, period of study, circulating variants, levels and sources of pre-existing immunity, as well as differences in vaccine efficacy [78, 80].

### Hybrid immunity in context of repeated exposures

As the number of exposures to SARS-CoV-2, either from vaccination, infection, or combinations of these increases, one factor that should be considered in trying to understand the cumulative effect of exposures on immunity and protection is whether the source (vaccine or virus) of the exposure matters. In the early phase of the pandemic, clinical studies on the 2-dose mRNA vaccine regimens showed similar or higher titers of antibodies in vaccinees when compared to convalescent controls [81, 82]. Subsequent epidemiological studies suggested that protection from infection was superior after vaccination than infection [83, 84]. However, more recent studies have shown the reverse, that protection from infection is greater from a previous infection than from vaccination alone [85–88]. This reversal may reflect the changing nature of the pandemic and the different therapies and modalities used to treat people with COVID-19, although the risks of poor outcomes from infection continue to far outweigh those from vaccination [89]. It should also be noted that recovery from natural infection has at least 2 inherent advantages over current SARSCoV-2 vaccines: a longer period of persisting antigen that can drive greater affinity maturation and breadth of MBCs [90], and the induction of an early, more robust and possibly more effective IgA mucosal response [91, 92].

As vaccination rates have increased, studies have shifted from considering effects from either infection or vaccination alone on immunity or protection to focusing on how the combination of infection and vaccination compares to vaccination or infection alone, although the latter is increasingly rare. Several large epidemiological studies have shown that protection from symptomatic infection is highest among people who were previously infected and vaccinated [76, 87, 93, 94]. A synergistic effect involving imprinted cellular responses may explain the enhanced magnitude of antibody responses and breadth for protection against emerging variants that has been described with hybrid immunity [95, 96]. However, the benefits from hybrid immunity may diminish as the number of vaccine doses increases. Studies have shown that infection followed by 1 dose of the mRNA vaccine induces higher frequencies of spike-specific plasmablasts and/or MBCs compared to 2 doses of vaccine alone [52, 59, 97]. However, as mentioned above, antibody and B-cell responses to a second vaccine dose did not increase over the first dose in people who were previously infected [57, 58, 62, 65]. Notably, differences in frequencies of spike-specific MBCs between uninfected and prior-infected diminished 2-3 months after vaccination [97], and by month 6 after vaccination, frequencies of spike-specific MBCs and MBC responses to variants of concern were similar between previously infected and uninfected vaccinees [52]. However, one advantage of hybrid immunity may be the level of somatic hypermutation, which at 3-4 months post-vaccination was higher in the prior-infected than uninfected group [52]. Other studies have shown similar outcomes [59, 95]; however, one caveat is that these analyses were performed at relatively early timepoints after vaccination.

The limited benefit of the second dose of an mRNA vaccine in previously infected individuals may be due to repeated exposure to antigen and/or the short time interval between doses. While repeated exposure to antigen under controlled conditions has been shown to induce potent, broad, and sustained immunity [98], these conditions are difficult to control and can lead to suboptimal responses. Antibodies from a previous exposure can mediate feedback inhibition by limiting antigen access during germinal center reactions [99], and repeated antigen exposure can create bottlenecks and restrict antibody diversity [66]. Diminished antibody responses to influenza vaccination have been linked to repeated vaccination and high pre-existing antibody titers [67, 68]. A similar mechanism may also be restricting B-cell responses to SARS-CoV-2 vaccines in people who have received passive administration of SARS-CoV-2 antibodies [100]. Furthermore, atypical MBCs, which are induced by SARS-CoV-2 infection and vaccination [30, 97], have been shown under conditions of chronic antigen stimulation to over-express inhibitory receptors and diminish their responsiveness to further stimulation [45]. In a recent study on malaria, it was proposed that while atypical MBCs are induced following exposure to the pathogen through infection or vaccination, repeated boosting favors atypical over conventional MBCs [101]. Thus, the outcome of MBC recall responses in the context of repeated exposures remains difficult to predict, posing challenges to the goals of generating sustained protective immunity against current and future SARS-CoV-2 variants and other pathogens [102, 103].

We recently evaluated the effect of infection on antibody and B-cell responses to a booster (third) dose of the mRNA vaccines in a cohort including people who were infected prior to or after vacci-nation or who remained uninfected throughout the 2-month study period [104]. Similar to other studies [34], we found that a third dose in uninfected individuals induced robust and broad antibody and B-cell responses that were increased in people infected post-boost yet muted in those who were infected prior to receiving their booster vaccine. The antibody and B-cell responses induced were most restricted in those individuals who received their vaccine closest to their time of infection. This abrogation of antibody and B-cell responses to a third dose of mRNA vaccine by recent SARS-CoV-2 infection is consistent with a recent report at 7 days post-vaccination [105], and the blunted responses observed after the second dose in previously infected people [57, 58, 62, 65].

Several possibilities could explain the unresponsiveness of B cells to a third vaccine dose after a recent SARS-CoV-2 infection. First, we considered anergy or exhaustion that has been described for CD21^lo^ B cells (reviewed in [54]), which are expanded during SARS-CoV-2 infection [18, 96, 97]. Under conditions of chronic activation, these CD21^lo^ B cells show signs of activation *in vivo* yet are refractory to further stimulation *ex vivo* [106], a phenomenon that has been termed post-activated anergy or exhaustion [107]. A hallmark of anergic B cells is failure to respond to BCR stimulation, as measured by calcium flux or phosphorylation of signaling molecules [108]. We focused on spleen tyrosine kinase (SYK) and the downstream phospholipase Cγ2 (pPLCγ2), 2 signaling molecules that undergo coordinated and rapid phosphorylation following BCR stimulation yet have profiles that distinguish different MBC subsets [31], and become dysregulated in severe cases of SARS-CoV-2 due to decreased expression of inhibitory receptors [109]. When we evaluated the response to stimulation among SARS-CoV-2 recently infected and uninfected individuals, we found that BCR-mediated signaling among spike-specific MBCs was higher at baseline in the individuals who were previously infected when compared to those who were uninfected, although levels were similar between the 2 groups at day 60 post-vaccination. Furthermore, we found that the fold difference in BCR signaling between baseline and day 60 was correlated with the interval between infection and vaccination [104].

The differences we observed in B-cell signaling between prior-infected and uninfected individuals were at baseline, prompting us to consider whether this reflected differences in phenotypes of spike-specific MBCs at this timepoint. While we did not find that baseline frequencies of spike-specific CD21^lo^ atypical MBCs differed between prior-infected and uninfected groups, we did find a significantly higher frequency of CD21^+^CD27^lo^ MBCs in the uninfected group [104]. Notably, these MBCs have been associated with a durable response to influenza vaccination [110], and more recently to SARS-CoV-2 vaccination [111, 112]. They are also similar in phenotype to the early MBC C6 that we found to correlate with primary immunization antibody responses [30] and depicted in Figure 2. In the recent study, we found that CD21^+^CD27^lo^ MBCs were less responsive to BCR stimulation than their CD21^+^CD27^+^ counterpart, providing an explanation for why baseline B-cell signaling was higher in the prior-infected group where CD21^+^CD27^+^ MBCs were enriched [104]. We do not have a mechanistic explanation for the differences in BCR signaling and there may be other factors contributing to the muted response of B cells to a third vaccine dose in people who were recently infected with SARS-CoV-2. Our findings do not address whether these differences were driven by infection per se or any source of repeated antigenic stimulation. Nonetheless, our findings may help provide guidance on when booster vaccines should be administered.

## CONCLUSIONS

The primary immune response to SARS-CoV-2 mRNA vaccines engages both plasmablasts and MBCs that can be evaluated simultaneously using high-dimensional flow cytometry. Given the heterogeneity of the responding B cells and their fluctuations over time, longitudinal studies that track immune responses should be performed using analytical tools that can allow for maximum uniformity and meaningful discovery. While there is a growing number of methods and algorithms being developed for projecting and clustering large sets of data, these applications are only useful if the flow cytometry is performed adequately. Beyond these considerations, there remains ongoing challenges of describing and discussing cluster-based complex datasets in ways that are concise and informative. Secondary immune responses to SARS-CoV-2 are best evaluated by considering exposures from both infection and vaccination. While it is clear that hybrid immunity generated from both infection and vaccination is superior to either alone, there is mounting evidence that repeated exposures may restrict antibody and B-cell responses. This evidence comes from studies that revealed muted immune responses and limited protection from a second dose of the primary 2-dose mRNA vaccines in people who were previously infected, and recent findings of a similarly muted response to booster vaccines. However, many of these observations have been based on small studies and clearly there is more to learn on the effect of timing between vaccine doses and the various factors that can impact immunity and protection against SARS-CoV-2 infection or re-infection.
